# The South African breast cancer and HIV outcomes study: Profiling the cancer centres and cohort characteristics, diagnostic pathways, and treatment approaches

**DOI:** 10.1371/journal.pgph.0002432

**Published:** 2023-10-24

**Authors:** Witness Mapanga, Oluwatosin A. Ayeni, Wenlong Carl Chen, Judith S. Jacobson, Alfred I. Neugut, Paul Ruff, Herbert Cubasch, Daniel S. O’Neil, Ines Buccimazza, Sharon Čačala, Laura W. Stopforth, Hayley A. Farrow, Sarah Nietz, Boitumelo Phakathi, Tobias Chirwa, Valerie A. McCormack, Maureen Joffe

**Affiliations:** 1 Faculty of Health Sciences, Strengthening Oncology Services Research Unit, University of the Witwatersrand, Johannesburg, South Africa; 2 Faculty of Health Sciences, DSI-NRF Centre of Excellence in Human Development, School of Clinical Medicine, University of the Witwatersrand, Johannesburg, South Africa; 3 Faculty of Health Sciences, Department of Medicine, Division of Medical Oncology, School of Clinical Medicine, University of the Witwatersrand, Johannesburg, South Africa; 4 Faculty of Health Sciences, Department of Radiation Oncology, University of the Witwatersrand, Johannesburg, South Africa; 5 National Cancer Registry, National Health Laboratory Service, Johannesburg, South Africa; 6 Faculty of Health Sciences, Sydney Brenner Institute for Molecular Bioscience, University of the Witwatersrand, Johannesburg, South Africa; 7 Herbert Irving Comprehensive Cancer Center, Vagelos College of Physicians and Surgeons, Columbia University, New York, NY, United States of America; 8 Department of Epidemiology, Mailman School of Public Health, Columbia University, New York, NY, United States of America; 9 Department of Medicine, Vagelos College of Physicians and Surgeons, Columbia University, New York, NY, United States of America; 10 South Africa Medical Research Council Common Epithelial Cancers Research Centre, University of Witwatersrand Faculty of Health Sciences, Johannesburg, South Africa; 11 Faculty of Health Sciences, Department of Surgery, University of the Witwatersrand, Johannesburg, South Africa; 12 Yale Cancer Center and Department of Medicine, Yale University, New Haven, CT, United States of America; 13 Department of Specialized Surgery, Inkosi Albert Luthuli Central Hospital, Durban and Ngwelezane Hospital, University of KwaZulu-Natal, Empangeni, KwaZulu-Natal, South Africa; 14 Departments of Surgery and Radiation Oncology, Grey’s Hospital, University of KwaZulu-Natal, Pietermaritzburg, KwaZulu-Natal, South Africa; 15 Charlotte Maxeke Surgical Breast Unit, Charlotte Maxeke Johannesburg Academic Hospital, Johannesburg, South Africa; 16 Faculty of Health Sciences, Department of Surgery, School of Clinical Medicine, University of Kwa-Zulu Natal, Durban, South Africa; 17 Faculty of Health Sciences, School of Public Health, University of the Witwatersrand, Johannesburg, South Africa; 18 Section of Environment and Radiation, International Agency for Research on Cancer, Lyon, France; Dow University of Health Sciences, PAKISTAN

## Abstract

The South African Breast Cancer and HIV Outcomes prospective cohort (SABCHO) study was established to investigate survival determinants among HIV-positive and HIV-negative SA women with breast cancer. This paper describes common and unique characteristics of the cancer centres and their participants, examining disparities in pathways to diagnosis, treatment resources and approaches adopted to mitigate resource constraints. The Johannesburg (Jhb), Soweto (Sow), and Durban (Dbn) sites treat mainly urban, relatively better educated and more socioeconomically advantaged patients whereas the Pietermaritzburg (Pmb) and Empangeni (Emp) sites treat predominantly rural, less educated and more impoverished communities The Sow, Jhb, and Emp sites had relatively younger patients (mean ages 54 ±14.5, 55±13.7 and 54±14.3 respectively), whereas patients at the Dbn and Pmb sites, with greater representation of Asian Indian women, were relatively older (mean age 57 ±13.9 and 58 ±14.6 respectively). HIV prevalence among the cohort was high, ranging from 15%-42%, (Cohort obesity (BMI ≥ 30 kg/m^2^) at 60%, self-reported hypertension (41%) and diabetes (13%). Direct referral of patients from primary care clinics to cancer centre occurred only at the Sow site which uniquely ran an open clinic and where early stage (I and II) proportions were highest at 48.5%. The other sites relied on indirect patient referral from regional hospitals where significant delays in diagnostics occurred and early-stage proportions were a low (15%- 37.3%). The Emp site referred patients for all treatments to the Dbn site located 200km away; the Sow site provided surgery and endocrine treatment services but referred patients to the Jhb site 30 Km away for chemo- and radiation therapy. The Jhb, Dbn and Pmb sites all provided complete oncology treatment services. All treatment centres followed international guidelines for their treatment approaches. Findings may inform policy interventions to address national and regional disparities in breast cancer care.

## Introduction

Breast cancer (BC) is the highest incident cancer, accounting for 11.7% of all cancers, is the leading cause of cancer death among women worldwide [1–7], and accounts for 6.6% of all cancer deaths globally [7]. Although the incidence is currently 88% higher in transitioned versus transitioning countries, it is still rapidly increasing in low-and-middle-income countries (LMICs) [8]. These LMICs contribute over 53% of all new global BC cases and about 62% of global BC mortality [9], though with great inter- and intra-country variation in reported incidence, morbidity, and mortality [8,10]. This is due to differences in population age and racial distributions, lifestyle risk factor prevalence, and availability of screening programs [11]. The sub-Saharan African region (SSA) has the highest breast cancer mortality rates globally, where the estimated 5-year survival of women diagnosed with breast cancer is a low 40% on average (albeit with large ranges between countries), as compared to 90% in the United States (US) [8,12]. For example, Western African Nigeria has an age-standardized mortality rate (ASR) of 25.9 per 100,000 versus the world average of 12.9 ASR. These variations reflect generally weak health infrastructure and resources, linked to lower country human development indices and varying levels of access to screening and early diagnosis services and cancer treatments [8,9]. By 2050, the prevalence of breast cancer in SSA is expected to double, and modifiable patient, community, and health system factors associated with high mortality rates must be identified and addressed [13].

South Africa (SA), an upper middle-income country, also has a rapidly increasing cancer burden and breast cancer is the most common cancer among women, accounting for almost 21% of all the female cancers [14]. Age-standardized incidence rates of breast cancer differ by ethnicity. The SA National Cancer Registry (SANCR) reported 2019 data at 88.05 per 100,000 for white women, 50.63 per 100,000 for Asian women, 46.53 per 100,000 for women of mixed ancestry, and 20.41 per 100,000 for black women [15,16]. Mortality rates for cancer are not routinely reported by the SA pathology-based SANCR and this motivated us to establish the South African Breast Cancer and HIV Outcomes cohort (SABCHO) study [6] at 5 tertiary cancer centers located in two Provinces of South Africa (Gauteng and KwaZulu-Natal). Summary findings include that the cohort bares a high HIV and noncommunicable diseases multimorbidity (MM) burden [17] and that 58% of the cohort is diagnosed with late-stage (III and IV) disease [18] and delays in initiating surgical treatments, chemotherapy, radiotherapy, and endocrine therapy occurred, particularly for women living less than 20 kilometres from the hospital or not speaking English [12]. At 3-years post diagnosis, the overall mortality rate was a high 47% for women with stage I-III breast cancer and 80.5% for women with stage IV disease, compared with just 10% in the United States (US) [19,20]. We showed that stage at diagnosis, HIV, multimorbidity and delays to initiating surgery as the first treatment all negatively impact overall survival [17,20–22].

As part of a larger sub-Saharan African cohort study investigating disparities in survival outcomes, improvements in early diagnosis and treatment were predicted to contribute to the largest increases in survival, with a combined absolute increase in survival of up to 22% when compared with the contributions of other factors such as HIV or aggressive breast cancer subtypes [19]. In our SABCHO cohort, breast cancer centre disparities were the predominant determinant of the stage at diagnosis and that patient disparities in socioeconomic status and knowledge and awareness of breast cancer were all modifiable factors that also impacted the stage at diagnosis [18]. In this paper, we attempt to describe these SABCHO patient and cancer center disparities in greater detail in the hope that they will inform national and ultimately regional policy interventions, aligned with global aspirations [23] to address inequities in cancer management.

## Methods

### Ethical statement

The SABCHO study was approved by the University of the Witwatersrand Human Research Ethics Committee (Approval Number: Ml50351, dated: 6th May 2015, recertified M1911203 dated 28 January 2020), the University of KwaZulu-Natal Biomedical Research Committee, BF080/15, and the Institutional Review Board of Columbia University (protocol number AAAQ1359, dated 1st January 2016).

### Study setting and data source

**Fig 1:**
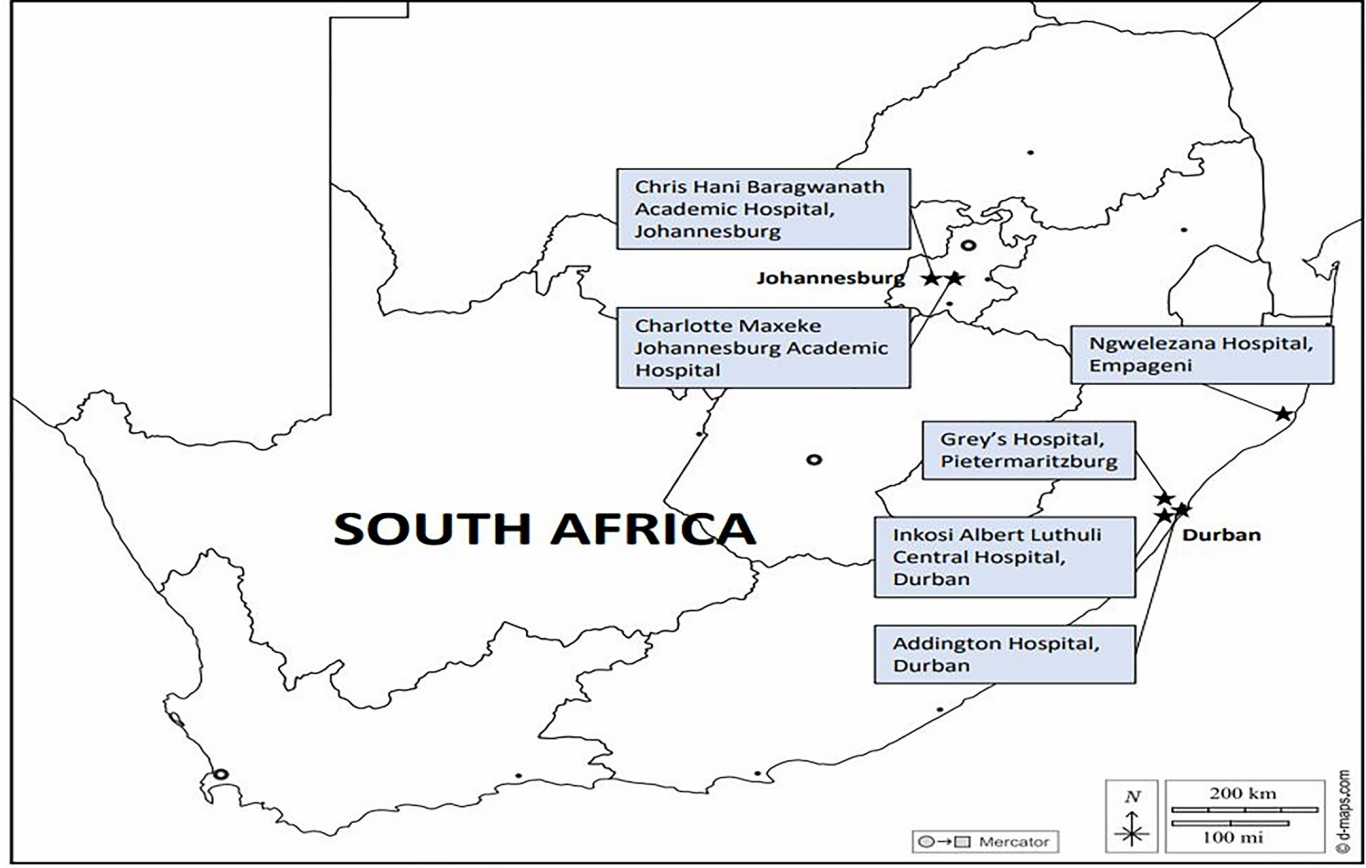
Hospitals participating in the SABCHO study. The source of the original map is D-Maps on https://d-maps.com/carte.php?num_car=4413&lang=en.

The majority (almost 84%) of the SA population, burdened with high levels of poverty, unemployment, and inequality [24,25], depend on the resource-constrained three-tier (primary, secondary, and tertiary/quaternary) public healthcare system which is predominantly funded by the government. Public cancer diagnostic and treatment services are provided at no cost to patients who do however bear transport and visit costs for hospital access [6]. The SABCHO cohort study [6] prospectively enrolled 3,497 women with newly diagnosed invasive breast cancers between 2015 and 2019 from two University of the Witwatersrand associated hospital sites located in Soweto, southern Johannesburg (Chris Hani Baragwanath Academic Hospital, (Sow)) and in central Johannesburg (Charlotte Maxeke Johannesburg Academic Hospital (Jhb)) and three University of KwaZulu-Natal associated hospital sites, Inkosi Albert Luthuli Central Hospital and its satellite Addington Hospital, in Durban (Dbn), Greys Hospital in Pietermaritzburg (Pmb) and Ngwelezane Hospital in Empangeni (Emp) (Fig 1. Hospitals participating in the SABCHO study (https://d-maps.com/carte.php?num_car=4413&lang=en) (Terms of use: https://d-maps.com/conditions.php?lang=en).

### Participants recruitment, inclusion criteria, and data collection

Women were eligible for recruitment in the SABCHO study if they were at adults (aged 18 or more years of age, enrolled between July 1, 2015, and December 31, 2019, newly diagnosed with stage I-IV invasive BC, had no prior history of cancer, and received their BC treatment at one of the tertiary/quaternary hospital sites. Participants were clinically staged at the time of diagnosis using the 7^th^ edition of the American Joint Committee on Cancer (AJCC).

Each participating hospital had dedicated study interviewers who collected sociodemographic characteristics, risk factors, comorbidities, and breast cancer clinical information knowledge and awareness of breast cancer and delay periods to diagnosis as previously described [6,17]. Some of the collected information and variables included the following: sociodemographic information (such as age, marital status, employment status, and level of education completed); behavioural factors (alcohol consumption, smoking); reproductive history (use of oral and injectable contraceptives, number of full-term pregnancies). Details of distances (and their calculations) from the place of residence to the place of diagnosis and other clinical variables have been reported previously [6,18].

A Barriers to Care questionnaire was used to assess awareness and knowledge of breast cancer (9 questions), full details of which have been previously reported [14]. The awareness and knowledge score (0–9) was calculated from summations of 1 (yes) and 0 (no) values assigned respectively for correct and wrong answers to questions and correct and incorrect agreements to statements. One-on-one, face-to-face interviews between the participants and a trained (multi-lingual) study interviewer were used to collect the awareness and knowledge of breast cancer information. Laboratory identifier codes recorded on the National Health Laboratory Service histopathology reports enabled confirmation of residential distance to diagnostic facility calculations using geographic information system (GIS) coordinates and determination of so-called direct and indirect pathways to diagnostic and treatment services, as previously described [14,26].

### Statistical analysis

The descriptive statistics (sociodemographic and risk factor characteristics of women with stages I-IV breast cancer for each hospital site) were performed using Stata version 17 (StataCorp Ltd, Texas, USA). Proportions across hospital sites were determined with crosstabs with significant differences indicated by Chi-square tests and presented as percentages. Using analysis of variance (ANOVA) method we further ascertained the mean/median differences across these hospital sites. All the figures were generated using Microsoft excel. Further thematic descriptions of the treatment sites were generated through in-depth interviews with doctors who are managing cancer patients in those sites.

## Results

### Brief characteristics of recruitment sites

Due to its catchment area and large population base, Sow was the highest (39%) recruiting site for the SABCHO study. It runs a weekly outpatient surgical clinic, where patients are received either ‘*directly’* (61%) from primary care clinics in Soweto or ‘*indirectly’* via secondary hospitals (39%) located within 65 km from Sow. The Sow hospital provided tertiary diagnostic and surgery services, but chemotherapy and radiation oncology treatments necessitated the transfer of patients to the Jhb site located about 20 km away (Table 1). The two Dbn site hospitals, Addington and Inkosi Albert Luthuli Central Hospital (Dbn) shared diagnostic and treatment services. Each site ran a weekly outpatient surgical breast clinic where for all but the Sow site, patients were seen by appointment; Sow was the only site that ran an open clinic, where appointment scheduling was not required.

**Table 1:** Summary SABCHO site characteristics and treatment approaches.

Tertiary academic hospital sites	Soweto (Sow)	Johannesburg (Jhb)	Durban (Dbn)	Pietermaritzburg (Pmb)	Empangeni (Emp)
**Patient residential demographics**	Soweto (3 million) **Urban**	Johannesburg East and Central (1.5 million) **Urban**	Durban Metropolitan (3.5 million) **Urban**	Western KwaZulu-Natal (3.5 million) **Urban / Rural mix**	Uthungulu, Umkhanyakude, Zululand (3 million) **Rural**
**Proportional site accrual to the SABCHO study (%)**	39	25	17	17	2
**Surgical tertiary site ease of participant access**	Weekly open clinic -no appointments	Weekly clinic -by appointment only	Weekly clinic -by appointment only	Weekly clinic -by appointment only	Weekly clinic -by appointment only
**Referral mode for diagnosis (%)** ^**A**^	61 direct 39 indirect	59 direct 41 indirect	26 direct 74 indirect	7 direct 93 indirect	12 direct 88 indirect
**Location for core biopsy facilities**	78% tertiary H 22% secondary H	90% tertiary H 10% secondary H	26% tertiary H 74% secondary H	7% tertiary H 93% secondary H	10% tertiary H 90% secondary H
**Main curative intent treatment approaches taking patient and health system constraints into account-stage I-II**	Mainly primary mastectomy + adjuvant systemic chemo, RT, endocrine Rx	Mainly primary mastectomy + adjuvant systemic chemo, RT, endocrine Rx	Mainly primary systemic treatment with adjuvant BCS (where eligible) or mastectomy + adjuvant RT endo	Mainly primary mastectomy, some BCS and adjuvant systemic chemo, RT, endocrine Rx	Treated at the Durban site according to Durban site protocols
**Main locally advanced treatment approaches -stage III**	Neoadjuvant systemic therapy then mastectomy if eligible, then, RT/endo	Neoadjuvant systemic therapy then mastectomy if eligible, then RT/endo	Neoadjuvant systemic therapy; BCS/mast if eligible, RT/endo	Neoadjuvant systemic therapy then mastectomy if eligible, RT/endo	Followed Durban site protocols as treated at the Durban site
**Palliative treatment approaches -stage IV**	Palliative chemo/ RT and endocrine Rx -surgery for bone mets if possible	Palliative chemo/ RT and endocrine Rx -surgery for bone mets if possible	Palliative chemo/endocrine -surgery where possible with bone metastases	Palliative chemo/some palliative surgery -and occasionally staging constraints caused inappropriate surgeries	Followed Durban site protocols as treated at the Durban site

^A^Direct referral means patient referral from the primary healthcare facility to the tertiary site; Indirect means referral from the primary care facility to the district secondary hospital to the tertiary treating site.

Abbreviations: HS = Health System; IHC = immunohistochemistry, Rx = treatment; RT = radiation treatment, endo = endocrine treatment.

### Demographic and socioeconomic characteristics of the participants at each site

#### Average age distribution

**Fig 2:**
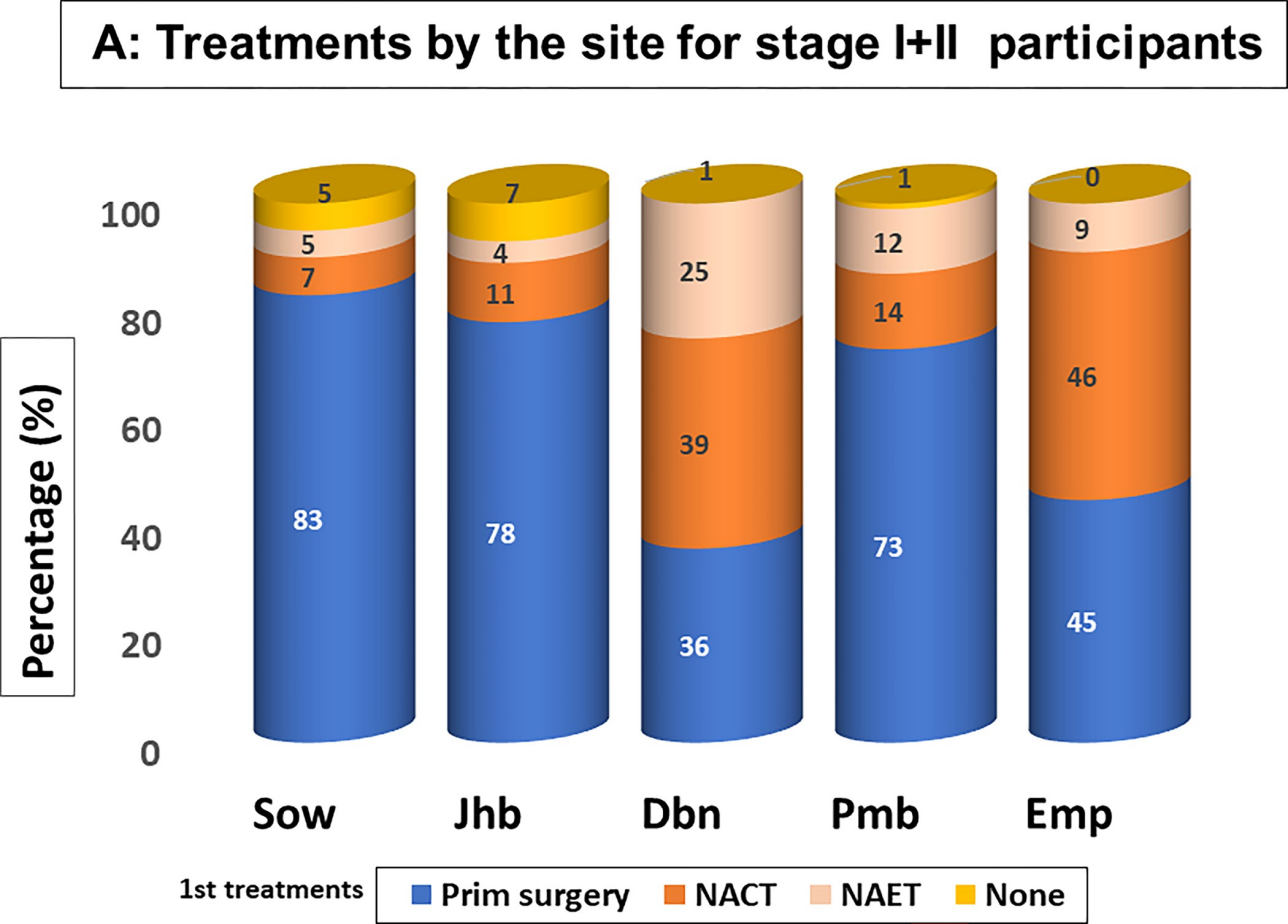


Sociodemographic, clinical, diagnostic and treatment characteristics of participants at each site are provided in Table 2 and grouped thematically in the supporting information files (S1–S5 Figs). Treatments for patients with the early and late-stage disease were described for each site (Figs 2 and 4). As shown in Table 2 (and S1 Fig) the Sow, Jhb and Emp sites had relatively younger patients (mean ages 54 ±14.54, 55±13.68 and 54±14.27 respectively), whereas the Dbn and Pmb sites had relatively older patients (mean age 57 ±13.98 and 58 ±14.55 respectively).

**Table 2:** Socio-demographic and risk factor characteristics of women with stages I-IV breast cancer in the SABCHO cohort by hospital site.

	Soweto	Johannesburg		Durban		Pietermaritzburg	Empangeni	Total	
	N (%)	N (%)		N (%)		N (%)	N (%)	N (%)	p-values
**Totals**	**1359 (*38*.*9)***	**870 (*24*.*9)***		**591 (*16*.*9)***		**604 (*17*.*3)***	**73 (*2*.*0)***	**3497 (100.00) **	
**Age at diagnosis in years**									
Average ± SD	55 ±14.54	54 ± 13.68		57 ±13.98		58 ± 14.55	54 ±14.27	**56 ±14.31**	**0.235**
21–39	217 (*16*.*0)*	142 (*16*.*3)*		69 (*11*.*7)*		70 (*11*.*6)*	13 (*17*.*8)*	**511 (14.6)**	
40–49	329 (*24*.*2)*	221 (*25*.*4)*		119 (*20*.*1)*		131 (*21*.*7)*	18 (*24*.*7)*	**818 (23.4)**	
50–59	333 (*24*.*5)*	207 (*23*.*8)*		136 (*23*.*0)*		140 (*23*.*2)*	18 (*24*.*7)*	**834 (23.8)**	
≥60	480 (*35*.*3)*	300 (*34*.*5)*		267 (*45*.*2)*		263 (*43*.*5)*	24 (*32*.*8)*	**1334 (38.2)**	
**Age at diagnosis in years**									
≤ 49 years	546 (*40*.*2)*	363 (*41*.*7)*		188 (*31*.*8)*		201 (*33*.*3)*	31 (*42*.*5)*	**1329 (38.0)**	**0.079**
≥ 50 years	813 (*59*.*8)*	507 (*58*.*3)*		403 (*68*.*2)*		403 (*66*.*7)*	42 (*57*.*5)*	**2168 (62.0)**	
**Ethnicity**									
Black	1261 (*92*.*8)*	666 (*76*.*5)*		294 (*49*.*8)*		479 (*79*.*3)*	71 (*97*.*3)*	**2771 (79.2)**	**0.183**
Asian	16 (*1*.*2)*	33 (*3*.*8)*		224 (*37*.*9)*		80 (*13*.*2)*	1 (*1*.*4)*	**354 (10.1)**	
White	24 (*1*.*7)*	126 (*14*.*5)*		51 (*8*.*6)*		24 (*4*.*0)*	1 (*1*.*4)*	**226 (6.5)**	
Mixed race	58 (*4*.*3)*	45 (*5*.*2)*		22 (*3*.*7)*		21 (*3*.*5)*	0 (*0)*	**146 (4.2)**	
**Residential distance from core biopsy facility**									
<20 km	991 (*72*.*9)*	440 (*50*.*6)*		367 (*62*.*0)*		262 (*43*.*0)*	4 *(5*.*5)*	**2064 (59.0)**	0.017*
20 to 59 km	304 (*22*.*4)*	407 (*46*.*9)*		155 (*26*.*3)*		80 (*13*.*0)*	14 *(19*.*2)*	**960 (27.5)**	
60 to 100 km	21 (*1*.*5)*	8 (*0*.*9)*		20 (*3*.*4)*		81 (*13*.*0)*	9 *(12*.*3)*	**139 (4.0)**	
>100 km	43 (*3*.*2)*	15 (*1*.*6)*		49 (*8*.*3)*		181 (*30*.*0)*	46 *(63*.*0)*	**334 (9.5)**	
**Highest level of education attained**									
Primary education and below	258 (*19*.*0)*	133 (*15*.*5)*		155 (*26*.*5)*		195 (*32*.*4)*	31 (*44*.*9)*	**772 (22.2)**	**0.104**
Secondary education and above	1096 (*81*.*0)*	728 (*84*.*5)*		429 (*73*.*5)*		407 (*67*.*6)*	38 (*55*.*1)*	**2698 (77.8)**	
**Employment**									
Employed	353 (*26*.*1)*	321 (*37*.*1)*		151 (*25*.*6)*		116 (*19*.*2)*	15 (20.6)	**956 (27.4)**	0.028*
Unemployed	735 (*54*.*2)*	343 (*39*.*6)*		400 (*67*.*9)*		380 (*62*.*9)*	56 (76.7)	**1914 (54.9)**	
Retired	267 (*19*.*7)*	202 (*23*.*3)*		38 (*6*.*5)*		108 (*17*.*9)*	2 (2.7)	**617 (17.7)**	
**Wealth index**									
1	187 (*13*.*8)*	109 (*12*.*5)*		109 (*18*.*4)*		248 (*41*.*0)*	46 (*63*.*1)*	**699 (20.0)**	0.042*
2	318 (*23*.*4)*	125 (*14*.*4)*		99 (*16*.*6)*		138 (*22*.*9)*	19 (*26*.*0)*	**699 (20.0)**	
3	322 (*23*.*6)*	189 (*21*.*7)*		116 (*19*.*6)*		69 (*11*.*4)*	4 (*5*.*5)*	**700 (20.0)**	
4	254 (*18*.*7)*	221 (*25*.*4)*		140 (*24*.*0)*		82 (*13*.*6)*	2 (*2*.*7)*	**699 (20.0)**	
5 (Wealthiest)	278 (*20*.*5)*	226 (*26*.*0)*		127 (*21*.*4)*		67 (*11*.*1)*	2 (2.7)	**700 (20.0)**	
**Family history of cancer**									
No	1166 (*87*.*5)*	716 (*84*.*3)*		462 (*79*.*8)*		520 (*89*.*4)*	64(*88*.*9)*	**2928 (85.8)**	**0.322**
Yes	166 (*12*.*5)*	133 (*15*.*7)*		117 (*20*.*2)*		62 (*10*.*6)*	8 (*11*.*1)*	**486 (14.2)**	
**Knowledge and awareness of breast cancer**									
Low/medium (0–6)	560 (41.6)	376 (43.5)		260 (44.2)		361 (59.8)	50 (68.5)	**1607 (46.2)**	**0.269**
High (7–9)	787 (58.4)	489 (56.5)		328 (55.8)		243 (40.2)	23 (31.5)	**1870 (53.8)**	
**Number of complete/full-term pregnancies (mean(sd))**									
	1263 3.1 (1.89)	791 3.0 (1.77)		528 3.2 (1.75)		564 3.7 (2.38)	68 3.9 (2.45)	**3214 (100)**	**0.684**
**Smoke cigarettes**									
Yes	131 (*9*.*7)*	138 (*15*.*9)*		89 (*15*.*1)*		72 (*11*.*9)*	3 (*4*.*1)*	**433 (12.4)**	**0.376**
No	1224 (*90*.*3)*	728 (*84*.*1)*		500 (*84*.*9)*		532 (*88*.*1)*	70 (*95*.*9)*	**3054 (87.6)**	
**Alcohol intake**									
Yes	294 (*21*.*7)*	167 (*19*.*3)*		101 (*17*.*1)*		101 (*16*.*7)*	9 (*12*.*3)*	**672 (19.3)**	**0.521**
No	1061 (*78*.*3)*	699 (*80*.*7)*		488 (*82*.*9)*		503 *(83*.*3)*	64 (*87*.*7)*	**2815 (80.7)**	
Body mass index (BMI)*									
<25	199 (*15*.*9)*	120 (*14*.*9)*	101 (*17*.*2)*		113 (*19*.*0)*		16 (*21*.*9)*	**549 (16.6)**	**0.344**
25–29.9	304 (*24*.*3)*	212 (*26*.*3)*	167 (*28*.*5)*		145 (*24*.*4)*		15 (*20*.*6)*	**843 (25.5) **	
≥30	748 (*59*.*8)*	474 (*58*.*8)*	318 (*54*.*3)*		336 (*56*.*6)*		42 (*57*.*5)*	**1918 (57.9) **	
**Diabetes**									
Yes	136 (*10*.*0)*	78 (*9*.*0)*	138 (*23*.*4)*		95 (*15*.*7)*		6 (*8*.*2)*	**453 (13.0)**	**0.873**
No	1219 (*90*.*0)*	788 (*91*.*0)*	451 (*76*.*6)*		509 (*84*.*3)*		67 (*91*.*8)*	**3034 (87.0)**	
**Hypertension**									
Yes	533 (*39*.*3)*	312 (*36*.*0)*	284 (*48*.*2)*		273 (*45*.*2)*		22 (*30*.*1)*	**1424 (40.8)**	**0.542**
No	822 (*60*.*7)*	554 (*64*.*0)*	305 (*51*.*8)*		331 (*54*.*8)*		51 (*69*.*9)*	**2063 (59.2)**	
HIV**									
Negative	1002 (*75*.*1)*	689 (*81*.*3)*	497 (*85*.*0)*		451 (*74*.*7)*		42 (*57*.*5)*	**2681 (77.8)**	**0.077**
Positive	332 (*24*.*9)*	159 (*18*.*7)*	88 (*15*.*0)*		153 (*25*.*3)*		31 (*42*.*5)*	**763 (22.2)**	
**HIV positives on ARVs at the time of breast cancer diagnosis**									
Yes	257 (*77*.*6)*	112 (*70*.*9)*	76 (*87*.*4)*		137 (*89*.*5)*		28 (*90*.*3)*	**610 (80.26)**	**0.298**
No	74 (*22*.*4)*	46 (*29*.*1)*	11 (*12*.*6)*		16 (*10*.*5)*		3 (*9*.*7)*	**150 (19.74)**	
**Histological diagnosis**									
Invasive ductal	1316 (*96*.*8)*	829 (*95*.*4)*	558 (*94*.*4)*		579 (*95*.*9)*		72 (*98*.*6)*	**3354 (95.9)**	**0.466**
Invasive lobular	39 (*2*.*9)*	39 (*4*.*5)*	31 (*5*.*3)*		21 (*3*.*4)*		1 (*1*.*4)*	**131 (3.8)**	
Other histological types	5 (*0*.*3)*	1 (*0*.*1)*	2 (*0*.*3)*		4 (0.7)		0 (0.0)	**12 (0.3)**	
**Stage at diagnosis**									
Stage I	69 (*5*.*1)*	77 (*8*.*9)*	19 (*3*.*2)*		35 (*5*.*8)*		0 (*0*.*0)*	**200 (5.7)**	0.011*
Stage II	590 (*43*.*4)*	255 (*29*.*3)*	198 (*33*.*5)*		190 (*31*.*5)*		11 (*15*.*1)*	**1244 (35.6)**	
Stage III	526 (*38*.*7)*	406 (*46*.*7)*	224 (*37*.*9)*		229 (*37*.*9)*		42 (*57*.*5)*	**1427 (40.8)**	
Stage IV	174 (*12*.*8)*	132 (*15*.*2)*	150 (*25*.*4)*		150 (*24*.*8)*		20 (*27*.*4)*	**626 (17.9)**	
**Receptor subtype**									
ER/PR+/HER2-	791 *(58*.*3)*	526 *(61*.*0)*	382 *(64*.*9)*		388 *(64*.*5)*		38 *(52*.*8)*	**2125 (61.0)**	**0.083**
ER/PR/ HER2+	277 *(20*.*4)*	131 *(15*.*2)*	80 *(13*.*6)*		73 *(12*.*1)*		12 *(16*.*7)*	**573 (16.5)**	
ER/PR-/HER2+ (HER2 Enriched)	80 *(5*.*9)*	64 *(7*.*4)*	45 *(7*.*6)*		35 *(5*.*8)*		9 *(12*.*5)*	**233 (6.7)**	
ER/PR/HER2- (Triple Negative)	208 *(15*.*3)*	142 *(16*.*5)*	82 *(13*.*9)*		106 *(17*.*6)*		*13 (18*.*1)*	**551 (15.8)**	
**Grade**									
1&2	811 *(61*.*0)*	459 (53.4)	295 *(61*.*8)*		384 *(67*.*5)*		30 *(50*.*0)*	**1979 (60.1)**	**0.723**
3	518 *(39*.*0)*	400 *(46*.*6)*	182 *(38*.*2)*		185 (32.5)		30 *(50*.*0)*	**1315 (39.9)**	

Missing data: Education levels 27, employment status 10, family history of BC 83, knowledge of BC 20, full-term pregnancies 283, smoking history 10, alcohol.

Intake 10, BMI 187, diabetes status 10, hypertension 10, HIV status 53, receptor subtyping 15, grade 203.

#### Racial distribution

The racial distribution of participants at each site largely mirrored regional distributions (Table 2). The Sow and Emp sites were mainly represented by Black patients (92.8% and 97.3% respectively); the Jhb site had the highest proportion of white participants (14.5%) and the Dbn and Pmb sites had relatively high representations of Asian Indian patients (37.9% and 13.2% respectively).

#### Participant residential distance and pathways to core biopsy diagnostic pathology services

As shown in Table 2 (and S2 Fig) a high proportion of Pmb and Empi site participants, (43% and 75% respectively) lived more than 60 kilometres from the diagnostic core biopsy facilities, whereas most women accessing the urban sites resided nearby the diagnostic centres (Sow (95%), Jhb (98%) and Dbn (88%), and these differences were significant (p = 0.017). For the Dbn, Pmb and Emp sites (Table 1), most of the participants (74%, 93% and 90% respectively) were referred from primary care facilities to regional secondary hospitals (‘*indirect route’*) where core biopsies were performed before being referred on to the tertiary sites for treatments.

#### Socioeconomic factors

As shown in Table 2 (and S3 Fig), the rural Pmb and Emp sites had the greatest proportion of extremely socioeconomically disadvantaged participants: (i) low household possession score proportions were respectively 75% and 95% compared with 61% for Sow, 49% for Jhb and 55% for the Dbn site; (ii) primary school or informal education only (a high 32% and 45% respectively compared with 19%, 16% and 27% for Sow, Jhb and Dbn sites), and (iii) 81% and 80% of participants were unemployed or retired compared with 74% Sow, 64% Jhb and 75% for the Dbn sites (albeit those unemployment rates were high for the whole cohort), and these differences were significant (p = 0.028).

#### Knowledge and awareness of breast cancer

As shown in Table 2 (and S4 Fig), the rural site participants had comparatively lower knowledge and awareness of breast cancer (only 40% for Pmb and 31% for Emp site had high knowledge and awareness scores compared with 58% for Soweto and 56% each for Jhb and Dbn sites). The rural sites also had the highest proportion of patients who self-reported delaying more than 3 months to seeking help following self-symptom detection (36% Pmb and 38% Emp compared with 26% Sow, 29% Jhb and 31% of Dbn site participants).

#### Multimorbidity burden

As shown in Table 2 (and S5 Fig), the multimorbidity burden (2 or more comorbidities) was high for the whole cohort. Obesity prevalence (BMI ≥ 30 kg/m^2^) ranged from 54% -60% across the sites; hypertension on treatment was self-reported in 48% and 45% of participants respectively for the Dbn and Pmb sites; for the Sow, Jhb and Emp sites the self-reported hypertension prevalence was relatively lower at 39%, 36% and 30% respectively. Self-reported diabetes on treatment prevalence was also extremely high among the Dbn and Pmb site participants at 23.4% and 15.7% respectively. HIV prevalence ranged from 15% to 42.5%.

### Description of diagnostic procedures for each site

Women with breast cancer were detected based on symptom self-presentation at primary care services. Population mammography screening is not available in SA. The Sow outpatient breast clinic was the only site to provide an open clinic where appointment scheduling for patients was not required, and all patients were seen. All other sites required appointment scheduling at the tertiary surgical breast clinics. Sow and Jhb tertiary hospital sites performed the majority (78% and 90% respectively) of triple assessment diagnostic procedures, including clinical staging assessments at the surgical breast clinics, mammography and sonography-guided core biopsies. In contrast, for the Dbn, Pmb and Emp sites, most participants received their core biopsy procedures in secondary regional hospitals (74%, 93% and 90% respectively) and were then referred to the tertiary sites for further staging and management.

#### Clinical stage at diagnosis

As shown in Table 2, overall, the cohort was diagnosed with late-stage breast cancer (only 5.7% with stage I, some 35.6% with stage II, (most were stage IIb cases), 40.8% with stage III disease and 17.9% with stage IV). Sow site had the highest proportion of patients with the earlier-stage disease (48.5% stage I and stage II combined), compared with 38.2% for Jhb, 36.7% for Dbn, 37.2% for Pmb and 15.1% for Emp site where no patients with stage I disease were diagnosed (albeit a small sample size), and these differences were significant (p = 0.011).

#### Breast Cancer Receptor subtype characteristics

The majority (95.9%) of the cohort was diagnosed with invasive ductal carcinomas, close to 4% with lobular carcinomas (no patients reported taking hormone-replacement therapy) and the rest with other histological types. The immunohistochemistry-determined receptor subtype distribution is presented in Table 2. Overall, 61.0% of the cohort had ER/PR+/HER2- (luminal A) breast cancer, 16.5% the ER/PR/HER2+ subtype (luminal B), 6.7% the ER/PR-/HER2+ (Her2-enriched) subtype and 15.8% the ER/PR-/HER2- (triple negative) subtype.

#### Treatment approaches at each site

**Fig 3:**
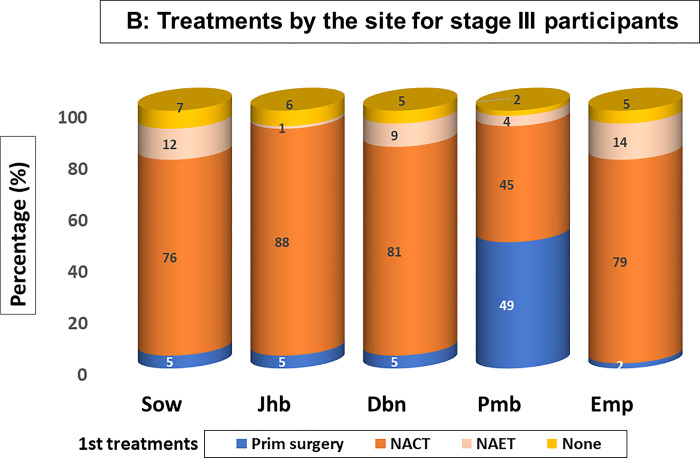


**Fig 4:**
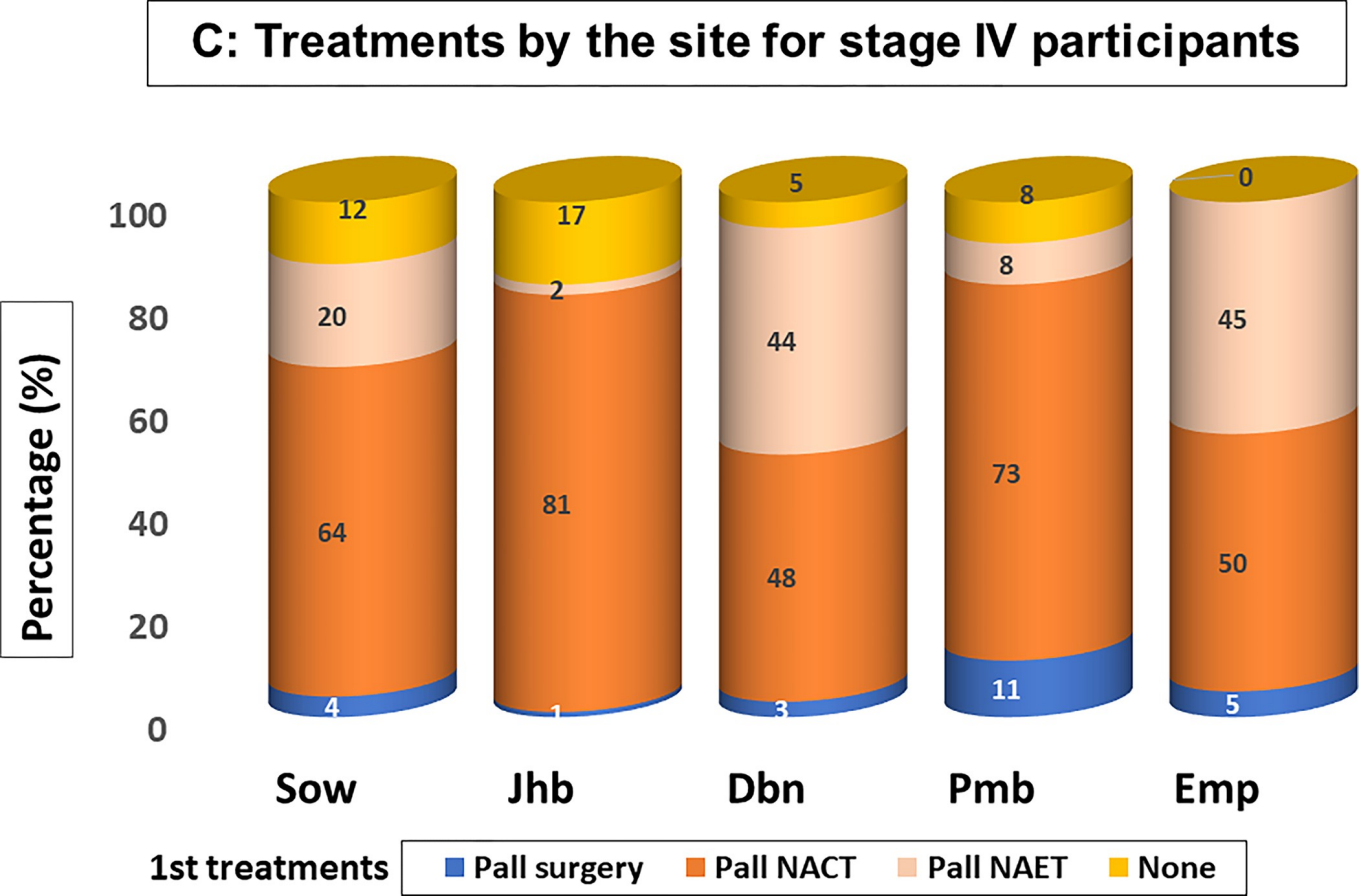


Treatments received at each site by patients with early stage, locally advanced and metastatic disease are summarised in Figs 2–4. A small percentages of patients received no treatment (average 4.4% of early- stage patients, 5.5% of patients with locally advanced disease and the 10.1% of patients with metastatic disease), results not shown, who either declined treatment or died prior to initiating planned treatments.

#### Surgical approaches

Treatment decisions were routinely made at weekly multidisciplinary tumour board meetings, based on South African, USA and European breast cancer management guidelines^-17^. For patients with an early-stage disease where curative intent was the goal, pragmatic approaches were applied at each site to consider both patient and site-specific health system arrangement complexities and constraints. The Soweto and Johannesburg sites were best resourced with surgeons and medical oncologists during the enrolment period, but radiation therapy resources were severely constrained. For early-stage disease (Table 2), (83.2% for Sow and 78% for Jhb), surgery was given as first treatments favouring mastectomies (77% for Sow and 64% for Jhb site) (Fig 2. Treatments by the site for stage I & II participants), followed by adjuvant chemotherapy and endocrine treatments, obviating the need for radiation therapy for the low-risk cases. The Dbn site (which also treated Emp patients), had limited surgical lists available, but had no constraints on chemotherapy, endocrine therapy and radiotherapy services. Only 35.5% (Dbn) and 45.5% (Emp) of early-stage patients received surgery as the first treatment. Surgery choices were governed by international guidelines and patient choices (49% mastectomies and 51% breast conserving surgery). For the Pmb site, surgery services were shared with referring regional hospitals, capable of doing simple mastectomies; 73.3% of patients received primary surgeries, 81% of which were mastectomies followed by adjuvant systemic treatments and radiation therapy where indicated.

#### Systemic and radiation oncology treatment approaches

The Soweto and Empangeni sites provided neither chemotherapy nor radiation oncology services at their centres; patients travelled to Johannesburg and Durban sites for treatment which added complexity to their management. The Johannesburg and Durban sites had adequate chemotherapy services (waiting times for treatment initiation seldom exceeded 60 days), but the Pietermaritzburg site had constrained chemotherapy services during the enrolment period, with delays of more than 60 days for chemotherapy initiation common, necessitating primary surgery for early-stage disease and more aggressive surgical approaches for locally advanced tumours.

For **locally advanced cases**, (Fig 3. Treatments by the site for stage III participants), as expected, neoadjuvant chemotherapy (NACT) to shrink tumours was the major treatment approach for most sites (Soweto 76%, Johannesburg 88%, Durban 81% and Empangeni 79%); subsequent adjuvant surgery for those that responded, radiation oncology and endocrine treatments were provided. For luminal A patients and the elderly, the Sow (12%) and Dbn sites (9% for Dbn and 14% for Emp patients) used neo-endocrine treatments (in accordance with international guidelines).

For women with **metastatic disease (**Fig 4. Treatments by the site for stage IV participants), palliative chemotherapy (64% Soweto, 81% Johannesburg, 48% Durban, 73% Pietermaritzburg and 50% Empangeni) and palliative primary endocrine treatments (20% Soweto, 2% Johannesburg, 44% Durban, 8% Pietermaritzburg and 45% Empangeni) were provided as the main treatment options, with primary surgery provided for patients with bone metastases fit enough to undergo surgery.

## Discussion

Major findings were that the cohort had a high burden of HIV and common noncommunicable diseases, which results in frailty, necessitating less aggressive treatment approaches [27,28], poorer responses to neoadjuvant chemotherapy and negatively impacts overall survival [17,21,27]. The sites differed in age and ethnicity distributions of patients managed and had differing pathways for patients to access diagnostic and cancer treatment centres. Referrals directly from primary care clinics to an open tertiary breast unit (no bookings needed), uniquely provided at the Sow site, resulted in earlier-stage proportions of patients diagnosed of breast cancer compared with the other sites. This suggests that direct referral to open tertiary centres are necessary components of intervention strategies required to address access barriers and to encourage earlier stage disease presentation in low-and-middle-income countries where mammography screening is not possible [14,16,29].

Treatment approaches at the sites were guided by multidisciplinary tumour boards and were generally in accordance with international guidelines. But there were inequities in availability of cancer treatment services, and for the cohort as a whole, we previously showed that more timely initiation of treatments is required [12]. Surgical service constraints at the Dbn site were accommodated with neoadjuvant systemic treatments (followed by adjuvant treatments) and mastectomy surgery over breast conserving surgeries at Sow and Jhb sites was chosen to avoid (where possible), constrained radiation oncology services [30]. The impact of such treatment approach adaptations on overall survival at the different sites requires investigation. Breast cancer management among rural patients is of particular concern requiring policy interventions to redress the inequities in cancer care. These were the most vulnerable and most challenging patients to treat in our cohort. They faced the greatest inequities in socioeconomic status, were the least educated, had the least knowledge and awareness of breast cancer and most were diagnosed with advanced disease. They had to travel substantial distances for diagnostic and treatment services, and we previously showed that long residential distances from treatment centres delayed initiating cancer treatments and negatively impacted overall survival [22]. Many of these patients received palliative primary surgical treatments (not in accordance with international guidelines), because of their difficulties in accessing chemotherapy and radiation treatments.

Our findings from the SABCHO cohort of disparities in sociodemographic characteristics, knowledge and awareness of breast cancer, pathways to diagnosis and treatments resources, agree with findings from other African and first world country settings reported in several recent review articles. Vast differences in average age at diagnosis reflect varying population age distributions and life expectancies of different African countries [8]. Sociodemographic and personal factors associated with vulnerable populations significantly impact the late stage at breast cancer diagnosis of the majority (two-thirds) of African patients and minority groups in high-income- countries. These include a general lack of knowledge and awareness of breast cancer and its symptoms, large distances to travel to cancer diagnostic and treatment facilities for rural women [31] low literacy rates, poverty associated with black race [32] particularly in rural African settings [33] and minority groups in high-income settings [34,35], lower levels of education, high unemployment, extremes of age, (very young and very old), fear of the disease and its treatments and associated stigma; unaffordability of treatments, religious and cultural issues to name but a few [8,11,13].

From a health services perspective, cancer diagnostic and treatment resources in SSA are generally inadequate. There are too few well-trained providers, inadequate diagnostic and treatment facilities and insufficient cancer drugs available [13]. The result is that too often patients experience long delays in accessing diagnostic services, compounded by misdiagnosis due to inadequately trained clinical staff and poor facility organization [36]. Where such services do exist, too often vulnerable patients in both SSA and in high-income countries (HICs) are unable to afford diagnostic and treatment services [34,35,37–39].

In SA, breast cancer diagnostic and treatment services, though constrained, are available to patients at no charge in public hospitals. The major lessons learned from our own study cohort are two-fold. Firstly, pathways to diagnostic facilities must be made to be as direct and as uncomplicated as possible for the patient. The open clinic option in contrast to patients needing to schedule appointments for their diagnostic visits may be one such strategy to facilitate their access. Secondly, guideline-concordant treatment approaches are available to oncologists to cope with service constraints. Mastectomy rather than breast conserving surgery can be favoured when radiation therapy services are limited and, in many instances neoadjuvant systemic approaches can be utilized to accommodate surgery constraints.

### Strengths and limitations of the study

The strength of this study is that it benefitted from a rich source of data collected from a large cohort of women with invasive breast cancer, managed in differing resource-constrained settings in South Africa. It gives an in-depth insight into the site and patient characteristics, differing patient and health system resources and infrastructure arrangements, pathways to diagnosis and treatments and successful adaptations to treatment resource constraints. Its major limitation is that very few participants were enrolled at the completely rural Empangeni site, given that our findings reveal potentially significant sociodemographic differences between urban and rural patients that may well influence overall survival in our cohort.

## Conclusion

This study provided an in-depth comparative description by oncology site of urban and rural patient sociodemographic and clinical characteristics, pathways to breast cancer diagnosis and treatment approaches. Findings may inform policy interventions to redress patient access barriers and inequities in service resources and infrastructure.

## Supporting information

S1 Fig Age at diagnosis. (TIF)

S2 Fig Residential distance, pathways and place for core biopsy. (TIF)

S3 Fig Socioeconomic factors. (TIF)

S4 Fig Breast cancer knowledge and self-delay period following symptom recognition. (TIF)

S5 Fig Multimorbidity burden. (TIF)
