# Supplementary figures and images for: The South African breast cancer and HIV outcomes study: Profiling the cancer centres and cohort characteristics, diagnostic pathways, and treatment approaches
Source: PLOS Glob Public Health. 2023 Oct 24;3(10):e0002432. doi: 10.1371/journal.pgph.0002432 (PMC10597516; doi:10.1371/journal.pgph.0002432)

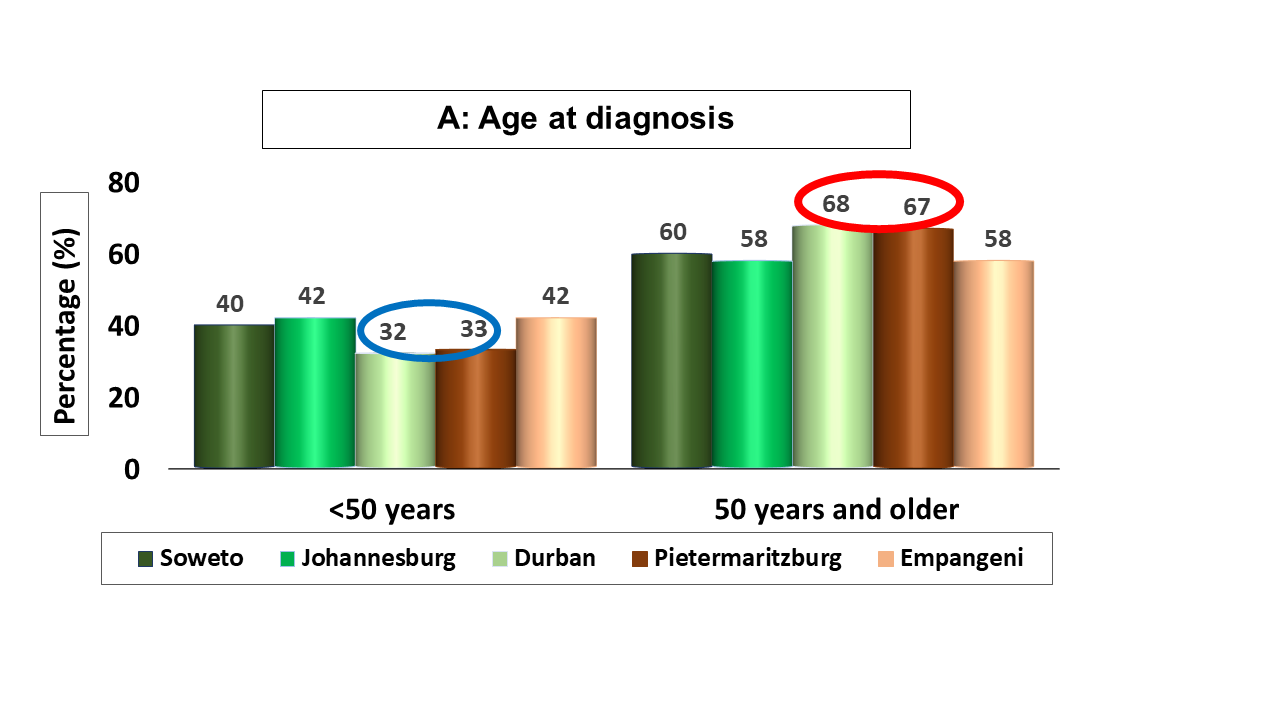

Supplement: S1 Fig — (TIF) [file pgph.0002432.s001.TIF]

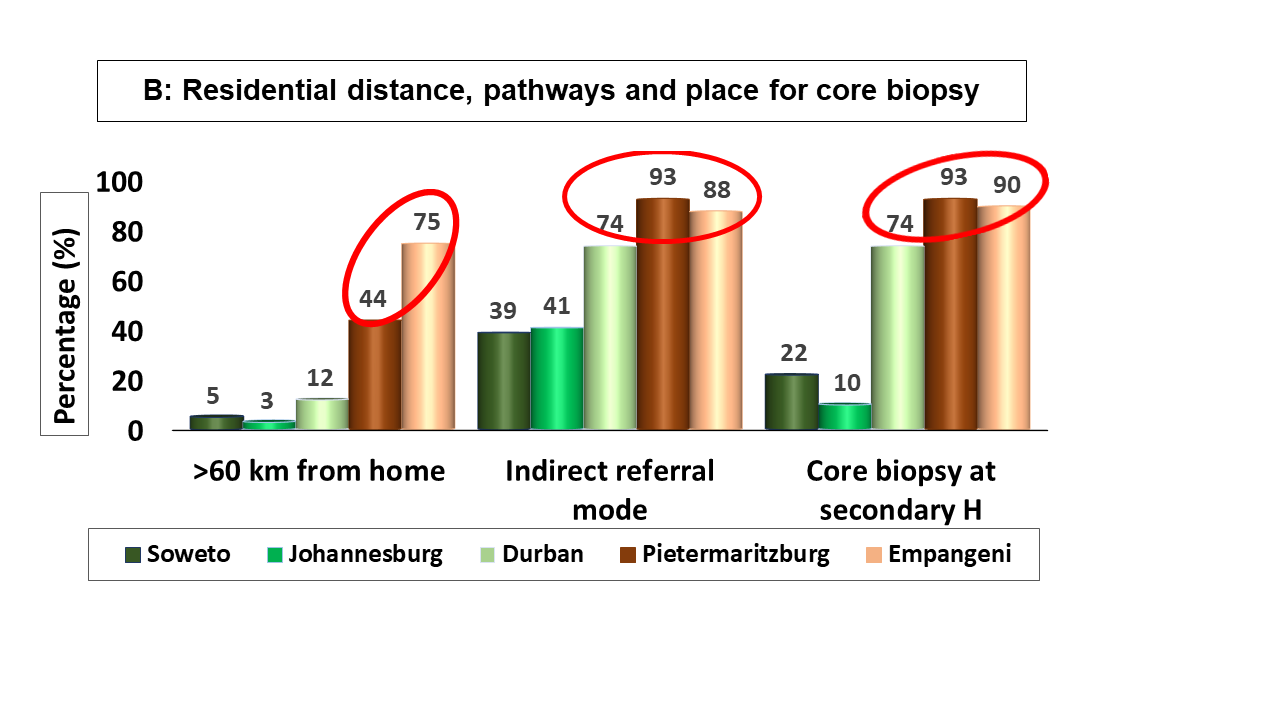

Supplement: S2 Fig — (TIF) [file pgph.0002432.s002.TIF]

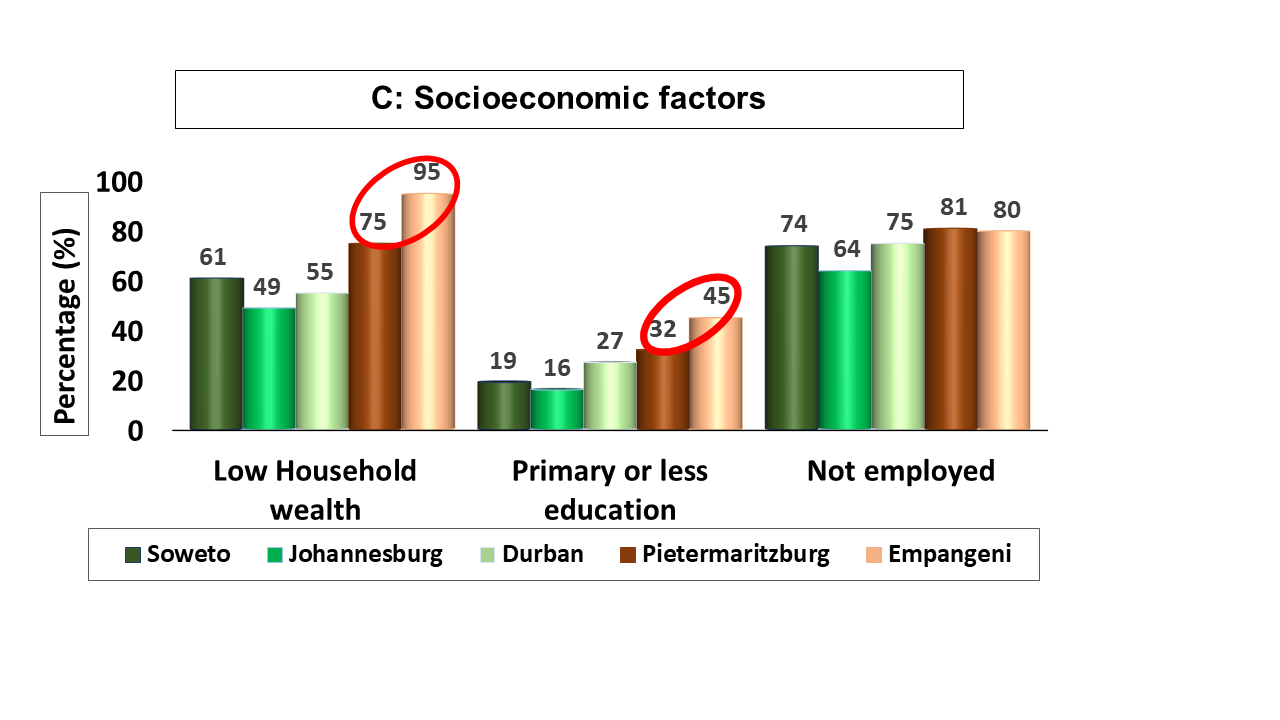

Supplement: S3 Fig — (TIF) [file pgph.0002432.s003.TIF]

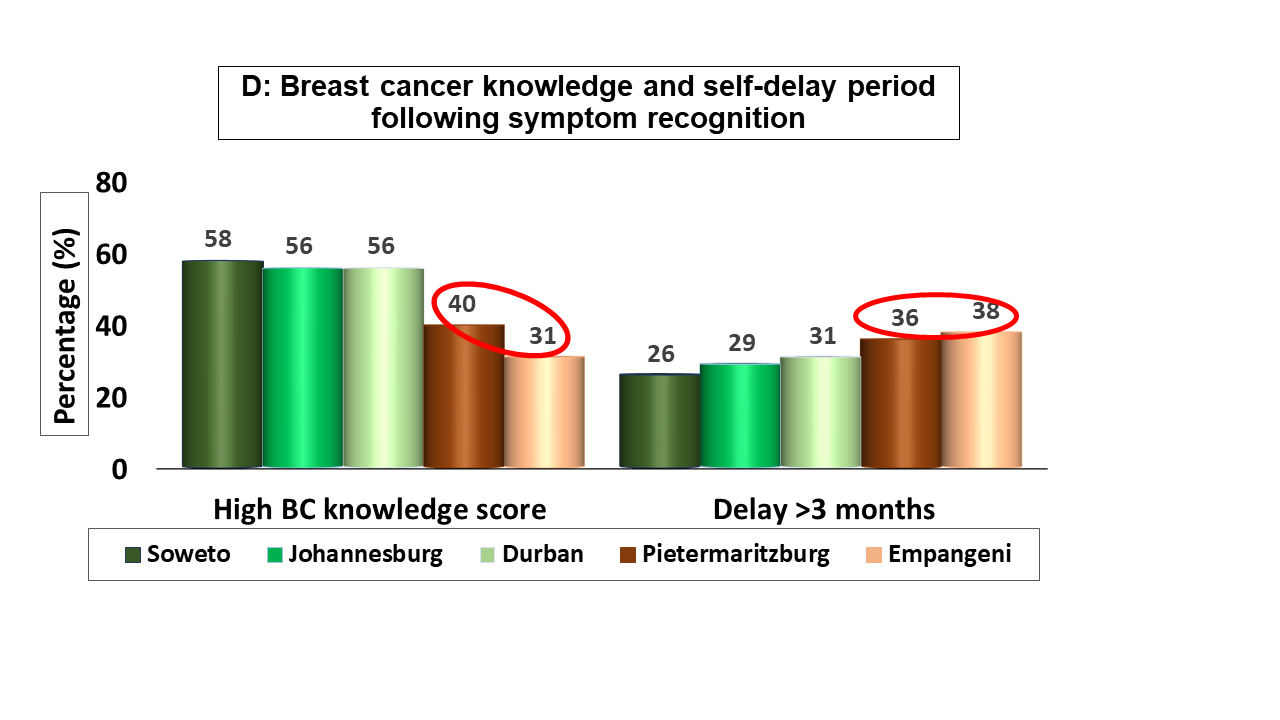

Supplement: S4 Fig — (TIF) [file pgph.0002432.s004.TIF]

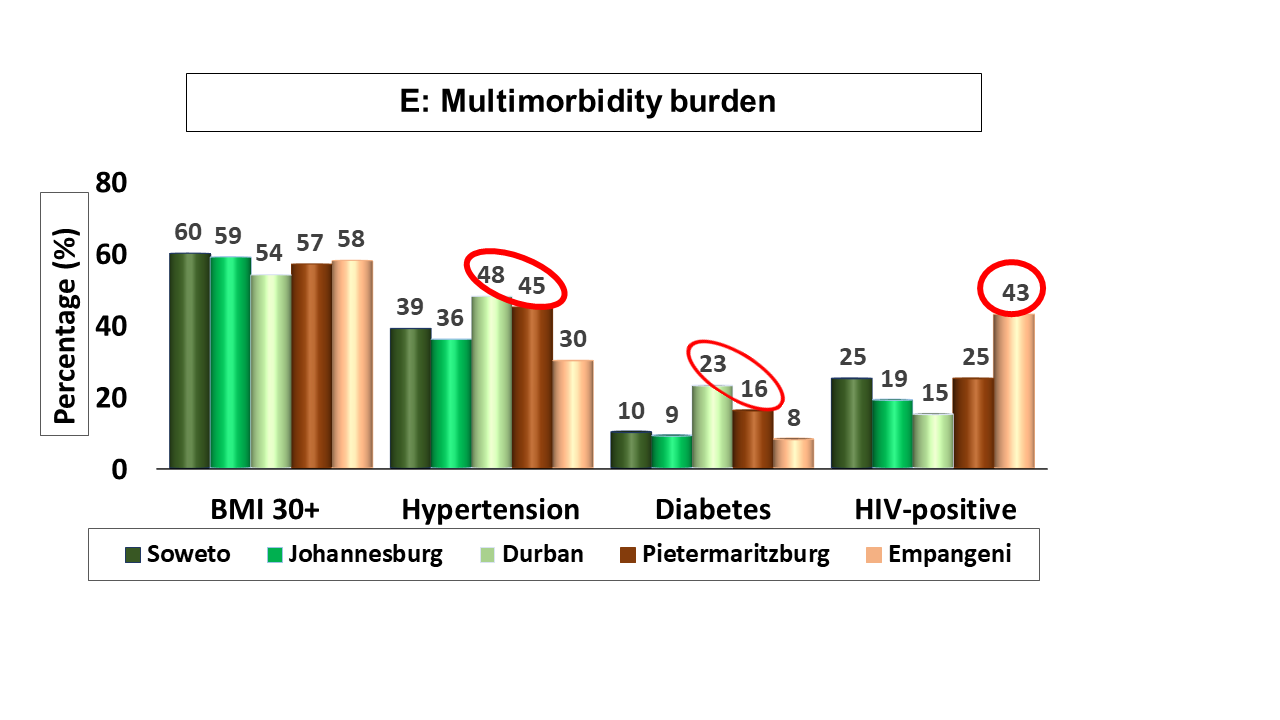

Supplement: S5 Fig — (TIF) [file pgph.0002432.s005.TIF]
